# Investigating adhesion of primary human gingival fibroblasts and osteoblasts to orthodontic mini-implants by scanning electron microscopy

**DOI:** 10.1038/s41598-024-68486-5

**Published:** 2024-07-30

**Authors:** Sarah Nadine Mirja Reimers, Martha Es-Souni, Sinan Şen

**Affiliations:** Department of Orthodontics, University Hospital of Schleswig-Holstein Campus Kiel, 24105 Kiel, Germany

**Keywords:** Cell biology, Medical research, Materials science

## Abstract

Miniscrews offer controlled anchorage and thus optimize tooth movement in orthodontic treatment. Nevertheless, failures such as soft tissue problems, instability due to loosening, partial osseointegration, or even device fracture can occur. While clinical technique can play a role in some of these problems, the manufacturer’s design and material choice influence how the implant interacts with the surrounding tissue. In some cases, the design and material may trigger unwanted bone and soft tissue responses. This in vitro study investigates how the implant surface affects cell adhesion and growth of human primary fibroblasts and osteoblasts on commercially available orthodontic TiAl6V4 miniscrews from three producers: tomas-pin SD N 08 (Dentaurum), OrthoEasy Pin (Forestadent), and Dual Top G2 (Promedia, Jeil Medical). Cell–implant interaction at the top, neck, and drilling part of the screws was assessed qualitatively by scanning electron microscopy. While both cell types adhered to and grew on all products, subtle differences in cell shape and spreading were detected, depending on the microstructure of the implant surface. This indicates that cell adhesion to implant surfaces can be controlled by manipulating the machining conditions.

## Introduction

**Figure 1:**
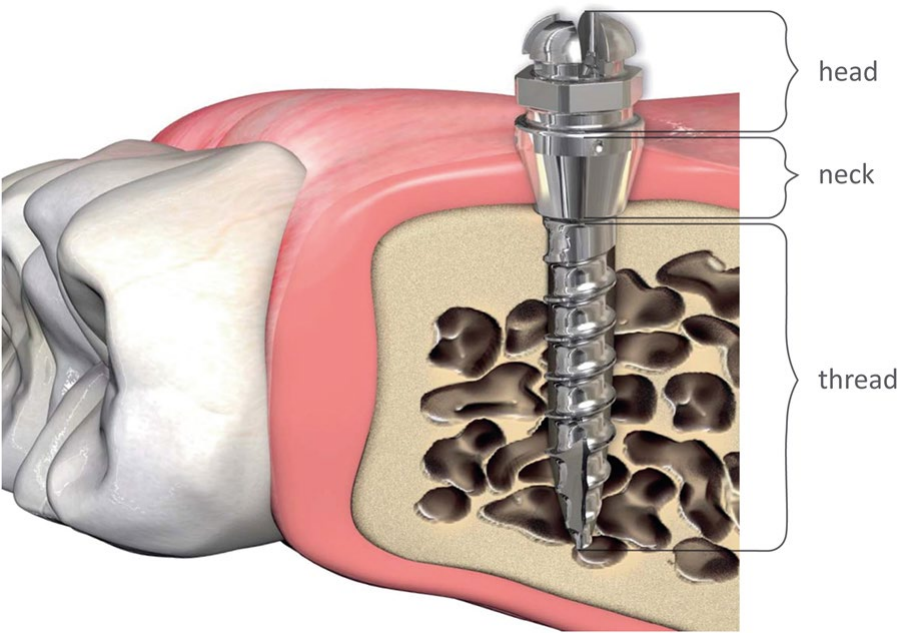
Structure of the miniscrew (tomas-pin shown here) and localization in the tissue. Modified from *tomas—the handbook*, courtesy of Dentaurum.

Orthodontic temporary attachment devices such as orthodontic mini-implants (OMIs) were first introduced in the 1990s^1,2^. They offer skeletal anchorage and control of tooth movement during orthodontic treatment^3,4^. In general, OMIs are composed of three parts: the head, the neck/collar, and the threaded body (Fig. 1). The head is used for dental attachment appliances, the neck provides close contact to the mucosa, and the body ensures endosseous fixation. OMIs reach from the oral cavity to the cortical and cancellous bone by traversing the mucosa, so are in contact with different tissues. Unlike permanent implants, OMIs are only mechanically retained to avoid in- and overgrowth of tissue, which may impede removal, but are in close contact to the neighboring tissue. OMIs are mostly precision-machined from titanium and its alloys. These titanium alloy materials are highly resistant to corrosion, and this resistance can be boosted by electrochemical treatments. They also have a relatively low elastic modulus, which matches the elastic modulus of bone, and a high specific strength/density. The biocompatibility of titanium and its alloys is well established and these are the most-used materials for OMIs. However, OMIs sometimes have negative side effects such as soft tissue inflammation, tissue overgrowth, loosening, and partial osseointegration^5–14^. Although clinical technique may explain some of these problems, the implant design (including the dimensions and surface micro-structure) affects the implant’s stability and tissue response, which influences treatment success. Particular attention has been devoted to the impact of surface topography on cell adhesion, growth, and detachment. Modifying the structure and dimensions of an implant surface may affect cell morphology and growth as well as inflammatory responses at the biointerface^15–17^. In particular, cell adhesion can be influenced by the roughness, chemical modification, or topography of the implant surface on a micro or nanometer scale^18–20^. This suggests that cell adhesion to implant surfaces can be controlled by manipulating the machining conditions. To overcome the risk of instability peaking in loss of the device, effort is made in order to design biocompatible implant surfaces with improved bone adhesion. This may be achieved by enhancing the surface roughness through sandblasted, large grit, acid-etched implant surface (SLA)^21–23^ as well as tuning the chemical surface composition with techniques such as Plasma ion implantation^24,25^ or UV treatment. For example, the treatment with UV light removes hydrocarbon, that settles on titanium surfaces over time. Consequently, the implant surface becomes superhydrophilic, promoting cell adhesion and proliferation^11,26,27^.

However, in this context, it is also important to mention, that miniscrews only maintain their stability by macroretention (primary stability) and not by osseointegration (secondary stability), so that easily removal of the screws is guaranteed. Therefore, the commercially available OMIs are only machine-processed and not SLA-processed^13,28^.

Most studies have been performed on model surfaces with well controlled topographical features. However, cells may behave differently on a commercially machined implant surface, and we investigate this in the present study. The OMIs investigated here also comprise a head, neck/collar, and threaded body, all of which have different shapes, dimensions, and mechanical characteristics, and these may affect cell behavior differently than model surfaces do^29^. The OMIs were made from the titanium alloy TiAl6V4, which is widely used for orthopedic and dental implants because it has good machinability, is resistant to corrosion, and is biocompatible^11,13,14^.

This study compares cell adhesion and surface structure of miniscrews in different areas and of different manufacturers. The aim is to find out to what extent cell adhesion is influenced by the surface structure of the miniscrews and whether typical complications can be influenced by further surface modification.

## Materials and methods

### Miniscrews

All three miniscrews (tomas-pin SD N 08 from Dentaurum, OrthoEasy Pin from Forestadent, and Dual Top G2 from Promedia, Jeil Medical) are made from a TiAl6V4 alloy that corresponds to the ASTM F 136 standard or DIN EN ISO 5832-3 (material number 3.7165)^30,31^. The components of the miniscrew alloy, according to these standards, are presented in Supplementary Table S1.

### Cell lines and cultivation

We examined the adhesion of two different cell lines to the miniscrews: primary gingival human fibroblasts from an extracted molar and a human osteoblast cell line (Human Osteoblasts, PromoCell, Heidelberg, Germany). Fibroblasts were cultivated in α-MEM (Minimum Essential Medium Eagle, Sigma-Aldrich Chemie GmbH, Hamburg, Germany) and osteoblasts (HOB) in osteoblast basal medium (Osteoblast Basal Medium, PromoCell, Heidelberg, Germany) supplemented with FCS and antibiotics as described in^32^. The cells were cultured at 37 °C, with 95% humidity and 5% CO_2_.

The screws were cleaned in 75% ethanol in an ultrasonic bath for 15 min and then sterilized at 121 °C for 15 min.

The study was approved by the ethics committee of the medical faculty of the Christian-Albrechts-University in Kiel (file number D457/11). All experiments were performed in accordance with relevant guidelines and informed consent was obtained from the participant who donated the extracted molar for the establishment of fibroblast cultures.

### Determination of the chemical composition by energy dispersive X-ray (EDX) analysis

Scanning electron microscopy (SEM) in combination with EDX has been used to assess the surface and chemical composition of materials^33^.

EDX revealed the relative frequency of the elements in the miniscrews and controlled the manufacturer specifications. Three screws per manufacturer were used for the EDX analysis. The calibration was done with a copper–aluminum sample, so it was only possible to determine elements in the screws.

### Microstructural analysis of the miniscrew surface by SEM

SEM has a significantly higher resolution than a light microscope^33^. A scanning electron microscope (XL 30CP, Philips, Amsterdam, Niederlande) was used to image the microstructure of the miniscrew surface. For imaging, the miniscrews were fixed on a sample plate and images were taken of the head, neck, and thread at 500× magnification.

### Qualitative assessment of cell adhesion by SEM

In the SEM images, cell adhesion to the different implants and screw areas could be assessed qualitatively. TC coverslips (Thermanox Plastic Coverslips, Thermo Electron LED GmbH, Langenselbold, Germany), were used as a positive control and poly(1,1,2,2-tetrafluoroethylene) (PTFE) was used as a negative control.

Miniscrew samples were loaded into two 24-well plates (one sample per well). Each 24-well plate contained two miniscrews from each manufacturer and two positive and negative controls. In the first 24-well plate, 9 × 10^4^ fibroblasts were seeded per well. In the second 24-well plate, 9 × 10^4^ osteoblasts were seeded per well. For feasibility reasons, 2 miniscrew samples per cell type were used in this pilot study for seeding. The plates were incubated for 24 h then the adhered cells were fixed and dehydrated for SEM investigation. In brief, all samples were washed three times with PBS, then fixed in 2.5% glutaraldehyde at 4 °C for 45 min. The cells were then rinsed with PBS and dehydrated through an ethanol series followed by hexamethyldisilazane (HMDS) (1 × 5 min 25% ETOH, 1 × 5 min 50% ETOH, 1 × 5 min 75% ETOH, 1 × 5 min 95% ETOH, 3 × 10 min 100% ETOH, 1 × 10 min 50% HMDS, 2 × 10 min 100% HMDS). Dried samples were stored in a desiccator for at least one night. Prior to SEM investigations, the samples were sputter coated with a 5-nm gold layer in a SCD500 Sputter Coater from BalTec AG. Micrographs of attached cells were taken to compare their morphology and number on the different sample surfaces.

## Results

**Figure 2:**
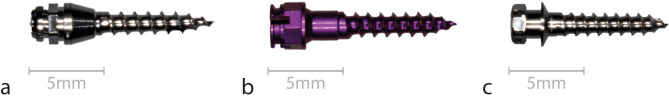
Examined miniscrews. (**a**) tomas-pin SD N 08 (Dentaurum, Ispringen, Germany). (**b**) OrthoEasy Pin (Forestadent, Pforzheim, Germany). (**c**) Dual Top G2 (Promedia, Siegen, Germany/Jeil Medical, Seoul, Republic of Korea).

The miniscrews used were photographed and are listed below (Fig. 2). The surface of the OrthoEasy Pin was anodized so had an oxide layer. According to the manufacturer, this is used for color coding to distinguish it from other variants and to improve primary stability.

### Determination of the chemical composition by EDX analysis

All elements were detected at similar concentrations in all screws. The EDX spectrum of miniscrew alloys is presented in Supplementary Fig. S1. An extra peak can be seen in the spectrum of the OrthoEasy Pins, which represents oxygen. This was expected as these screws have an oxide layer. An additional peak was detected at 0.26 keV for all products, which represents carbon and indicates surface contamination from the environment.

### Microstructural analysis of the miniscrew surface by SEM

**Figure 3:**
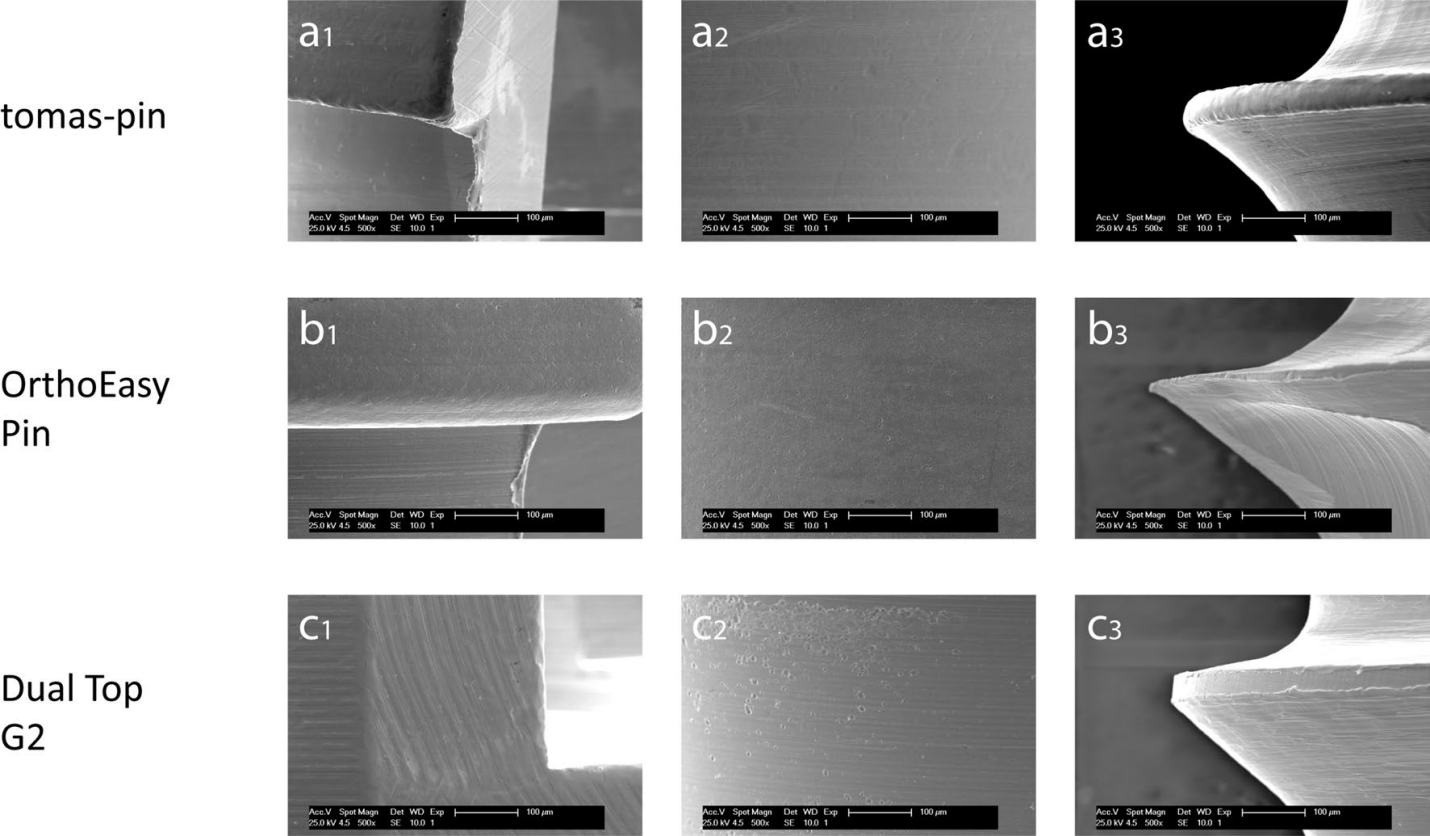
Surface texture. Scanning electron micrographs of the miniscrew surface at 500× magnification. tomas-pin—(**a**_**1**_) Head: excess substance at the edge with fine-cut facets in the cross slot. (**a**_**2**_) Neck: irregular indentations on a smooth surface. (**a**_**3**_) Thread: fine continuous groove structure at the flanks; rounded cutting edge. OrthoEasy Pin—(**b**_**1**_) Head: excess substance at the edge with fine-cut facets. (**b**_**2**_) Neck: punctiform indentations on a smooth surface. (**b**_**3**_) Thread: fine sectional groove structure at the flanks; sharp cutting edge. Dual Top G2—(**c**_**1**_) Head: defined edges without excess substance, but with various indentations; partially wide-cut facets. (**c**_**2**_) Neck: fine continuous groove structure overlaid with punctiform indentations. (**c**_**3**_) Thread: fine sectional groove structure at the flanks; flattened cutting edge.

To compare the implant surfaces, scanning electron micrographs were taken at 500× magnification (Fig. 3). Most of the heads had irregular edges and all had scratches and grooves from the finishing processing (Fig. 3a1,b1,c1). The necks differed significantly in their microstructure. While some were smooth (tomas-pin and OrthoEasy Pin), others had a finely grooved structure (Dual Top G2) while others had point-shaped indentations (OrthoEasy Pin and Dual Top G2) (Fig. 3a2,b2,c2). All the threads had a grooved structure. These grooves were continuous in some OMIs (tomas-pin) and limited to sections in others (Dual Top G2 and OrthoEasy Pin). There were also significant differences in the cutting edges of the screws—some had sharp edges while others had rounded or flattened edges (Fig. 3a3,b3,c3).

### Qualitative assessment of cell adhesion by SEM

**Figure 4:**
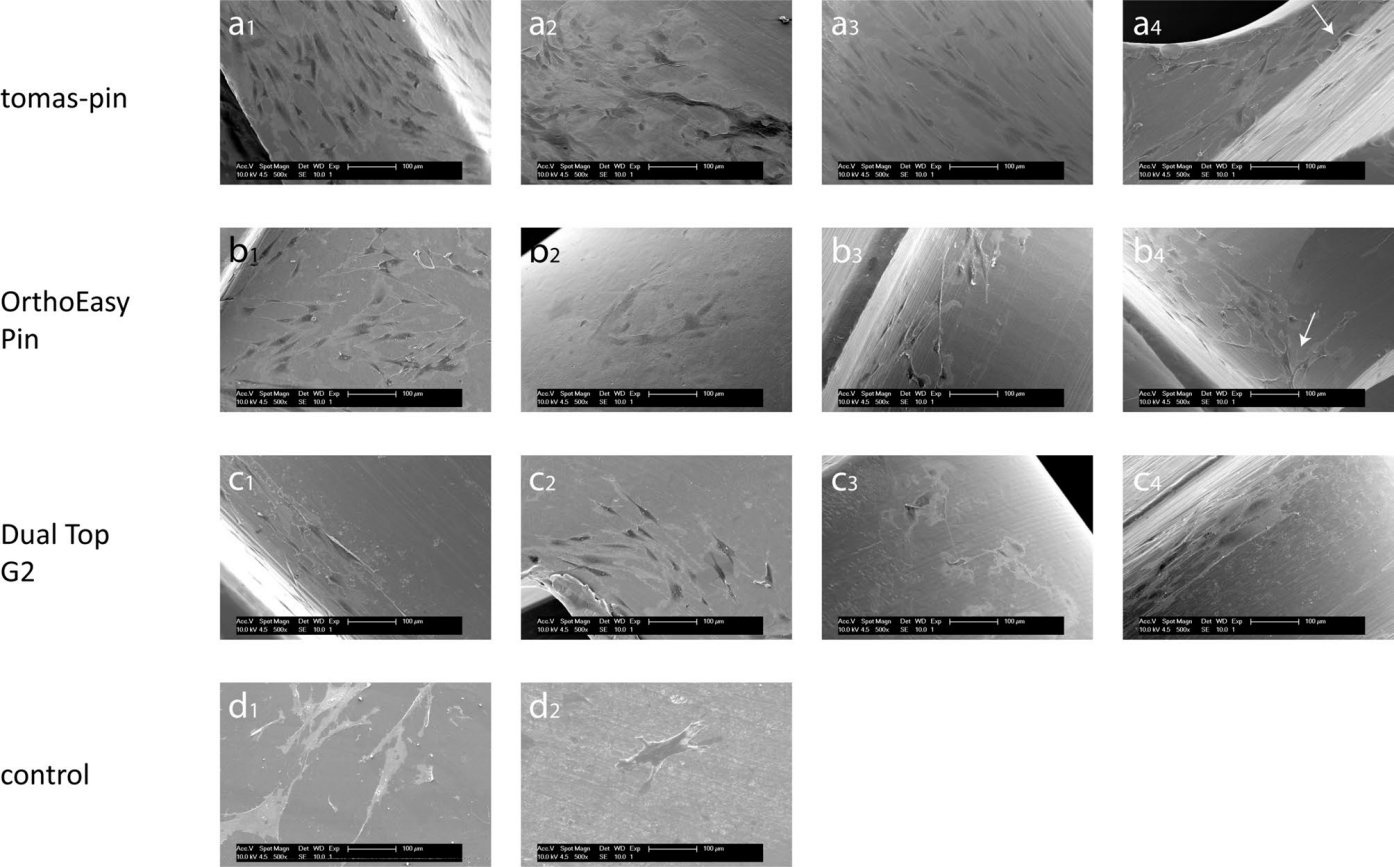
Fibroblast adhesion to miniscrews and controls. Scanning electron micrograph of fibroblast adhesion to screws and controls at 500× magnification. tomas-pin—(**a**_**1**_) Head: spindle-shaped cells. (**a**_**2**_) Neck: moderately elongated cells with polygonal shape. (**a**_**3**_) Thread: spindle-shaped cells oriented along the grooves. (**a**_**4**_) Thread (apex): spindle-shaped cells aligned along the grooves and with numerous protrusions (arrow). OrthoEasy Pin—(**b**_**1**_) Head: polygonal and spindle-shaped cells. (**b**_**2**_) Neck: moderately elongated cells with polygonal shape. (**b**_**3**_) Thread: spindle-shaped cells. (**b**_**4**_) Thread: spindle-shaped cells, the alignment along the grooves is particularly evident at the edge of the indentations (arrow). Dual Top G2—(**c**_**1**_) Head: spindle-shaped cells with orientation along the grooves. (**c**_**2**_) Head: polygonal and spindle-shaped cells. (**c**_**3**_) Neck: moderately elongated cells with polygonal shape. (**c**_**4**_) Thread: spindle-shaped cells with orientation along the grooves. control sample—(**d**_**1**_) TC coverslips (positive control): polygonal and spindle-shaped cells with numerous protrusions. (**d**_**2**_) PTFE (negative control): moderately elongated and spherical cells.

**Figure 5:**
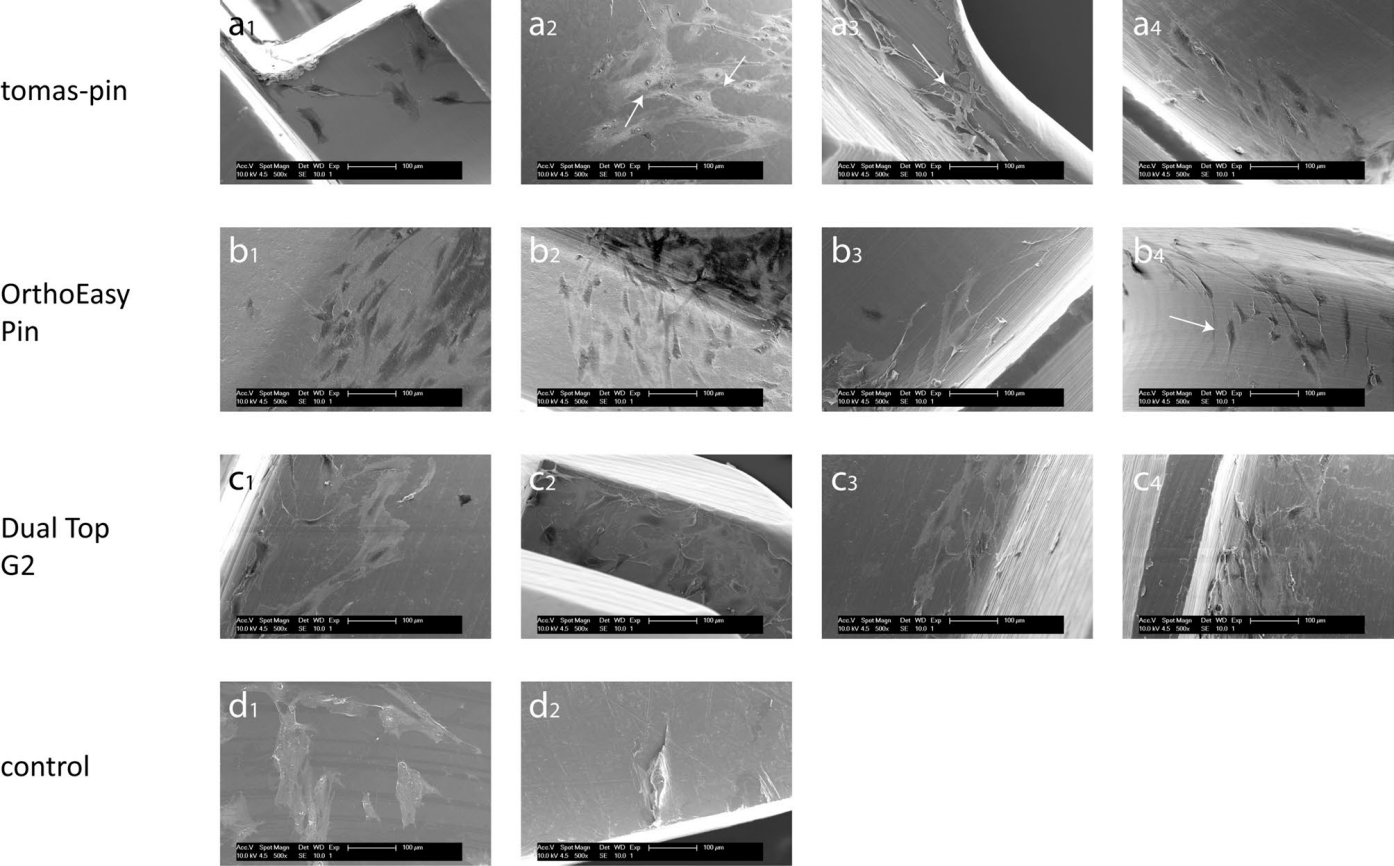
Osteoblast adhesion to miniscrews and controls. Scanning electron micrograph of osteoblast adhesion to different screw areas and controls at 500× magnification. tomas-pin—(**a**_**1**_) Head: polygonal cells with protrusions. (**a**_**2**_) Neck: polygonal cells with pronounced protrusions and recognizable cell nuclei (arrows). (**a**_**3**_) Thread: polygonal cells with pronounced protrusions; good networking of the cells is visible (arrow). (**a**_**4**_) Thread: polygonal and spindle-shaped cells oriented along the grooves. OrthoEasy Pin—(**b**_**1**_) Head: polygonal and spindle-shaped cells. (**b**_**2**_) Neck: polygonal cells with pronounced protrusions. (**b**_**3**_) Thread: polygonal and spindle-shaped cells with pronounced protrusions oriented along the grooves. (**b**_**4**_) Thread: polygonal and spindle-shaped cells aligned along the fine longitudinal grooves and along the overlying transverse grooves (arrow). Dual Top G2—(**c**_**1**_) Head: polygonal cells with protrusions. (**c**_**2**_) Head: polygonal cells with pronounced protrusions. (**c**_**3**_) Neck: polygonal cells with protrusions. (**c**_**4**_) Thread: polygonal and spindle-shaped cells. control samples—(**d**_**1**_) TC coverslips (positive control): spindle-shaped and polygonal cells with numerous extensions; high cell density. (**d**_**2**_) PTFE (negative control): moderately stretched, spherical cells; only isolated cells.

For the qualitative assessment of cell adhesion to the screws, scanning electron micrographs were taken (Figs. 4, 5). Fibroblasts and osteoblasts adhered to all sections of all OMIs. No sections were detected without cells attached.

TC coverslips were used as a positive control to illustrate the cell shape under optimal adhesion conditions. The cell density was high on these coverslips; fibroblasts were elongated and spindle-shaped, while osteoblasts were flat and polygonal shaped. PTFE was used as a negative control to observe the cell shape under poor adhesion conditions. Very few cells adhered to PTFE, and were either rounded or with very limited spreading.

Similar to the positive control conditions, fibroblasts were predominantly spindle-shaped and osteoblasts were predominantly polygonal shaped when adhered to the miniscrews. However, the shape of the cells was affected by the implant surface. In grooved areas (such as on the screw thread), both cell types were more spread out and spindle-shaped and orientated themselves parallel to the grooves. On smooth surfaces (such as the implant neck), osteoblasts were predominantly polygonal shaped and fibroblasts were only moderately spread out; spreading was more pronounced on the thread. The cells generally attached to the screw surface and extended numerous protrusions.

No differences in cell adhesion were detected between the different miniscrews. The OrthoEasy Pin was the only screw with an oxide layer, but this did not seem to affect cell adhesion to the implant surface. Isolated spherical-shaped osteoblasts and fibroblasts were observed on all miniscrews. Overall, cell density appeared higher on grooved surfaces than on smooth surfaces. However, cell density could not be quantified from SEM images.

## Discussion

This SEM study showed that the shape and surface microstructure differed between miniscrews. However, there were also similarities between the implants, such as the groove structure on the thread. Both osteoblasts and fibroblasts showed extended cell bodies on all miniscrews, indicating successful cell adhesion. Cells adhered to and grew on all areas of the implants and no qualitative differences in cell growth or adhesion were observed between the products. Cell adhesion to the implant surface was also not affected by an oxide layer.

However, the implant microstructure had subtle effects on cell shape and spreading. For example, cells oriented along the grooves. We also found that cells were less dense on smooth surfaces, and that the cells appeared to prefer a surface with microstructure, such as the screw threads. This suggests that cell adhesion can be controlled by modulating the implant surface.

It would be interesting to explore to what extent cell adhesion of the surrounding tissue would be beneficial for mini-implantation. Cell adhesion to the screw head is clearly undesirable since the head only serves to attach the orthodontic elements and thus protrudes freely into the oral cavity without tissue contact. Adhesion of cells to the head may be co-responsible for an overgrowth of connective tissue. This is a common complication of miniscrew implants^7,34^.

Fibroblast adhesion to the neck area was observed for all tested miniscrews and may be considered a desirable effect, since a tight fit to the mucosa reduces the risk of biofilm formation on the interface thus preventing infection and loosening^35,36^. We observed this in all the miniscrews we examined. In fact, the mucosa forms a 'biological seal', which creates a biological and physiological barrier around the implant. This mucosal barrier consists of two zones: the junctional epithelium and the connective tissue. Regarding the junctional epithelium, epithelial cells are connected to the implant surface via an internal basal lamina and hemidesmosomes. In the zone of the connective tissue, collagen fibres run parallel to the implant surface. They cannot adhere but are in contact with the implant via a sticky effect due to a high content of glycosaminoglycan and thus offer trauma-resistant adhesion to the gingiva^37,38^. Rough surfaces in this case would be rather undesired, as they lead to an increase in biofilm formation^39–41^. With their smooth surface in the neck area, the mini screws we examined seem to provide a good surface for the tight fit of the mucosa and for avoiding infections.

Osteoblast adhesion to the thread can increase implant stability by promoting bone attachment to the thread^36^. However, this can also promote partial osseointegration, making implant removal more complicated later on and increasing the risk of breakage^28,42^. For explanation, the miniscrews obtain their secondary stability through the attachment of bone to the thread^36^ while osseointegration does not occur due to the smooth surface^28^. Osseointegration, would result in a structural and functional connection between bone and implant, which would not allow relative movement between bone and implant^11,37,43^. So, osseointegration is clearly undesirable, since the implant should be easily removed^13,28^.

Several studies have investigated the appearance of machined and rough surfaces, as well as osteoblast cell adhesion under scanning electron microscopy (SEM)^44,45^.

Osseointegration is promoted on rough surfaces^11^, so the surfaces of the miniscrews we examined seem to represent a good compromise: the surface is microstructured enough for successful osteoblast adhesion, but osseointegration is largely prevented by the relatively smooth surface.

## Conclusion

This study shows that surface structuring of the miniscrews has an influence on the cell adhesion behavior. In some areas of the miniscrews the manufacturers used similar surface structuring, while in other areas the microstructuring differed considerably between the manufacturers. The fact that a generalized adhesion of cells was evident over the entire area of all miniscrews suggests that further surface modification of mini-implants may be beneficial in terms of avoiding typical complications. The question to which extent cell adhesion to the respective areas of the miniscrews is beneficial and what the corresponding surface modification could look like leaves room for further future studies.

### Supplementary Information

Supplementary Information.

## Data Availability

The datasets generated during and/or analyzed during the current study are available from the corresponding author on reasonable request. Additional data is also included in the supplementary information.
