# Supplementary material for: Investigating adhesion of primary human gingival fibroblasts and osteoblasts to orthodontic mini-implants by scanning electron microscopy
Source: Sci Rep. 2024 Jul 30;14:17475. doi: 10.1038/s41598-024-68486-5 (PMC11289300; doi:10.1038/s41598-024-68486-5)
Supplement: Supplementary file 1 — Supplementary Information. [file 41598_2024_68486_MOESM1_ESM.pdf]

Supplementary Information

Investigating adhesion of primary human gingival fibroblasts and osteoblasts to orthodontic mini-implants by scanning electron microscopy

Tables

	Ti	Al	V	Fe	O	C	N	H
**mass fraction (%) acc. to manufacturer information (acc. to DIN ISO 5832-3 / ASTM F 136)**	rest	5,5-6,75 / 5,5-6,50	3,5-4,5	max. 0,3 / 0,25	max. 0,2 / 0,13	max. 0,08	max. 0,05	max. 0,015 / 0,012

Tab. S1: Alloy components of the miniscrews according to the manufacturer information

EDX diagrams

tomas-pin

a

OrthoEasy Pin

b

Dual Top G2

c

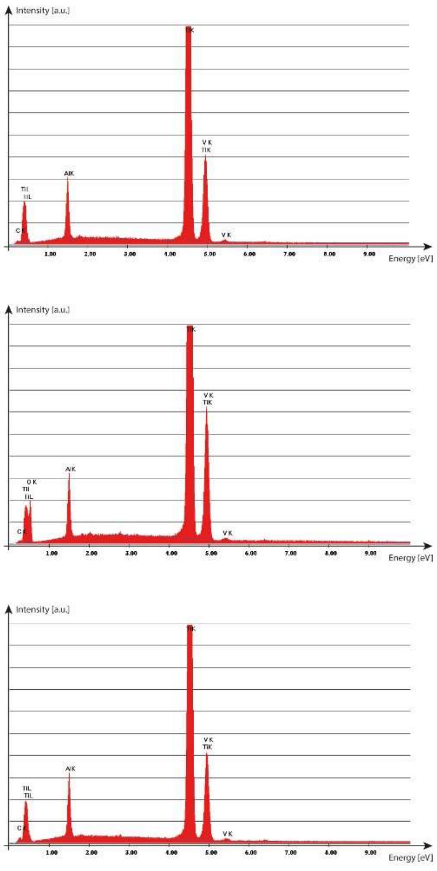

Fig. S1: EDX spectrum of the alloys of the mini screws
Main peaks of the K-shell, isolated indication of the secondary peaks of the L-shell

- **a** tomas-pin: Peak at 0.26keV represents carbon
- **b** OrthoEasy Pin: Peak at 0.50keV represents oxygen from the oxide layer, peak at 0.26keV represents carbon
- **c** Dual Top G2: Peak at 0.26keV represents carbon
